# The Combination of Novel Targeted Molecular Agents and Radiation in the Treatment of Pediatric Gliomas

**DOI:** 10.3389/fonc.2013.00110

**Published:** 2013-05-10

**Authors:** Tina Dasgupta, Daphne A. Haas-Kogan

**Affiliations:** ^1^Department of Radiation Oncology, University of California San FranciscoSan Francisco, CA, USA

**Keywords:** pediatric gliomas, low-grade glioma, BRAFV600E, PI3K/AKT/mTOR, angiogenesis inhibitors, bevacizumab, lenalidomide

## Abstract

Brain tumors are the most common solid pediatric malignancy. For high-grade, recurrent, or refractory pediatric brain tumors, radiation therapy (XRT) is an integral treatment modality. In the era of personalized cancer therapy, molecularly targeted agents have been designed to inhibit pathways critical to tumorigenesis. Our evolving knowledge of genetic aberrations in pediatric gliomas is being exploited with the use of specific targeted inhibitors. These agents are additionally being combined with XRT to increase the efficacy and duration of local control. In this review, we discuss novel agents targeting three different pathways in gliomas, and their potential combination with XRT. BRAF is a serine/threonine kinase in the RAS/RAF/MAPK kinase pathway, which is integral to cellular division, survival, and metabolism. Two-thirds of pilocytic astrocytomas, a low-grade pediatric glioma, contain a translocation within the BRAF gene called KIAA1549:BRAF that causes an overactivation of the MEK/MAPK signaling cascade. *In vitro* and *in vivo* data support the use of MEK or mammalian target of rapamycin (mTOR) inhibitors in low-grade gliomas expressing this translocation. Additionally, 15–20% of high-grade pediatric gliomas express BRAF V600E, an activating mutation of the BRAF gene. Pre-clinical *in vivo* and *in vitro* data in BRAF V600E gliomas demonstrate dramatic cooperation between XRT and small molecule inhibitors of BRAF V600E. Another major signaling cascade that plays a role in pediatric glioma pathogenesis is the PI3-kinase (PI3K)/mTOR pathway, known to be upregulated in the majority of high- and low-grade pediatric gliomas. Dual PI3K/mTOR inhibitors are in clinical trials for adult high-grade gliomas and are poised to enter studies of pediatric tumors. Finally, many brain tumors express potent stimulators of angiogenesis that render them refractory to treatment. An analog of thalidomide, CC-5103 increases the secretion of critical cytokines of the tumor microenvironment, including IL-2, IFN-γ, TNF-α, and IL-10, and is currently being evaluated in clinical trials for the treatment of recurrent or refractory pediatric central nervous system tumors. In summary, several targeted inhibitors with radiation are currently under investigation in both translational bench research and early clinical trials. This review article summarizes the molecular rationale for, and the pre-clinical data supporting the combinations of these targeted agents with other anti-cancer agents and XRT in pediatric gliomas. In many cases, parallels are drawn to molecular mechanisms and targeted inhibitors of adult gliomas. We additionally discuss the potential mechanisms underlying the efficacy of these agents.

## Introduction

Recent, exciting advances in pediatric oncology are revealing the molecular underpinnings of pediatric brain tumors (Nakamura et al., 2007), as well as important genetic differences between pediatric and adult central nervous system (CNS) malignancies (Gilheeney and Kieran, 2012). As brain tumors remain the most common solid malignancy of childhood, numerous molecularly targeted strategies are under investigation to decrease recurrence, prolong survival, and minimize toxicity in children treated with brain tumors. This article summarizes three of the major pathways targeted by novel inhibitors in the clinic, with a particular focus on low-grade gliomas in children.

## BRAF

BRAF is a cytosolic serine-threonine kinase and, together with A-Raf and C-Raf, is a member of the RAF family of kinases. BRAF is activated by receptor tyrosine kinases in the cell membrane and is a major regulator of the MEK/MAPK cascade, which regulates cell differentiation, invasion, dedifferentiation, and proliferation (McCubrey et al., 2012).

**Figure 1:**
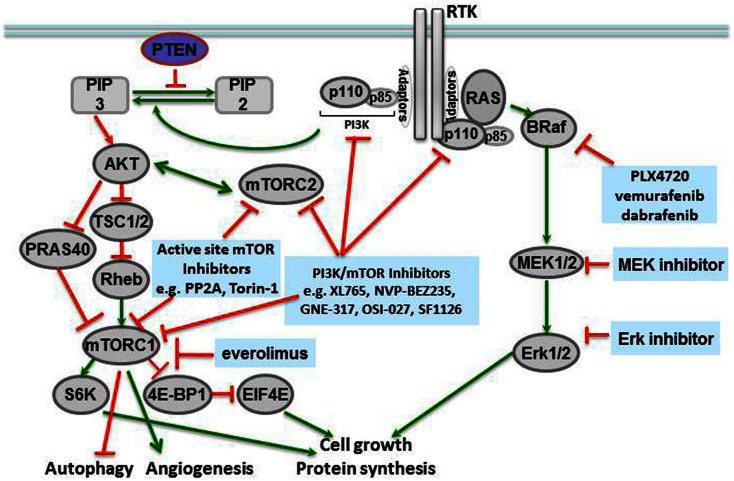
**Novel targeted agents and the pathways inhibited in solid tumors**.

Molecular profiling of tissues from different human cancers has shown that mutations of the BRAF gene at locus 1799 (amino acid 600) are the second most common mutations in human cancers, found in 40% of papillary thyroid carcinomas and 40–60% of melanomas (McCubrey et al., 2012). In this mutation, a valine in the activation segment of the enzyme is replaced with a negatively charged glutamic acid, mimicking a phosphorylation of the active site, and interacting with the P loop. The mutation causes constitutive activation of the BRAF kinase as well as downstream players in the signaling cascade (Figure 1). The prevalence of this mutation has led to the development of specific inhibitors against BRAF V600E, including vemurafenib (PLX4032, pre-clinical analog PLX4720) (Chapman et al., 2011) and dabrafenib, both of which have shown remarkable efficacy in BRAF V600E-mutated metastatic melanoma in Phase III clinical trials (Hauschild et al., 2012), and are discussed further below.

The activating BRAF V600E is found in 10–25% of grade 2–4 pediatric gliomas (Schiffman et al., 2010). In one study of 27 pediatric grade I and II pediatric brain tumors, the frequency of BRAF V600E mutation was queried using an oligonucleotide microarray, and 14 of 31 tumors (40%) were found to encode the BRAF V600E mutation (Dougherty et al., 2010). Anatomically, BRAF V600E mutations have been associated with extracerebellar pilocytic astrocytomas (instead of infratentorial ones) (Schindler et al., 2011). In some cases (five of seven in this series), these oncogenic BRAF mutations were associated with homozygous deletions of CDKN2A (Schiffman et al., 2010). There continues to remain controversy about the prognostic significance of the BRAF V600E mutation in pediatric gliomas. In a study of 198 pediatric low-grade gliomas, a BRAF V600E mutation was associated with poorer outcomes and decreased progression-free survival in patients, though the poorest survival was predicted by a midline anatomic location (Horbinski et al., 2012).

Another genetic aberration in BRAF in addition to BRAF V600E point mutations that leads to increased kinase activity in low- and high-grade pediatric brain tumors is a fusion protein of KIAA1549:BRAF that is found in the majority of pilocytic astrocytomas. The significance of this fusion is not well understood (Schiffman et al., 2010; Ida et al., 2012; Lin et al., 2012). Several studies suggest that, unlike the BRAF V600E mutation, the KIAA1549:BRAF fusion is associated with an infratentorial location for pilocytic astrocytomas (Horbinski et al., 2010; Lin et al., 2012). One recent study of 51 pilocytic astrocytomas suggests that the presence of a KIAA1549:BRAF fusion should be associated with a pilocytic classification, while a BRAF V600E mutation is more prevalent in non-pilocytic gliomas (Tian et al., 2011). There is some evidence that in pilocytic astrocytomas, the fusion protein is associated with a favorable outcome (Hawkins et al., 2011), while another series did not find any correlation with the BRAF fusion protein and disease progression (Lin et al., 2012).

The prevalence of oncogenic BRAF V600E mutations in several solid tumors has led to the development of anti-cancer agents that specifically inhibit BRAF V600E. PLX4032 or vemurafenib (F. Hoffman-La Roche AG) was the first inhibitor developed to demonstrate preferential activity against the BRAF V600E mutant protein versus the wild-type protein. In the BRIM3 trial, vemurafenib has shown remarkable activity against BRAF V600E metastatic melanoma, with 80% of patients showing either partial or complete remission, 74% reduction in disease progression or death, and a survival advantage over patients treated with dacarbazine alone, the standard of care (Chapman et al., 2011). Vemurafenib was subsequently FDA-approved for the treatment of BRAF V600E-mutated metastatic melanoma. Dabrafenib has also prolonged progression-free survival and overall survival when compared to dacarbazine in patients with BRAF V600E mutated melanoma in the BREAK3 trial (Hauschild et al., 2012). There is clinical evidence that dabrafenib also crosses the blood brain barrier, which supports its use in neuro-oncology (Long et al., 2012). Both dabrafenib and vemurafenib are remarkably well tolerated, with fatigue and photosensitivity being the major side effects. A small proportion of metastatic melanoma patients has developed a secondary skin cancer called keratoacanthoma while receiving BRAF V600E inhibitors, but these are considered to be treatable lesions with local therapy (excision or radiation), and some lesions have even disappeared with discontinuation of therapy (Falchook et al., 2012).

Excitingly, pre-clinical data in intracranial xenografts of pediatric gliomas suggest a role for BRAF V600E inhibitors in the treatment of pediatric gliomas. Recently published *in vivo* murine data suggest that BRAF inhibitors may be effective in high-grade gliomas expressing the BRAF V600E mutation (Nicolaides et al., 2011). This efficacy of PLX4720 (the pre-clinical analog of vemurafenib) has also been seen in a xenograft flank tumor mouse model of BRAF V600E-mutated pediatric pilocytic astrocytoma (our unpublished results). Furthermore, in an intracranial xenograft model of BRAF V600E-mutated glioma, mice were randomized to receive vector alone, BRAF inhibitor (PLX4720) alone, radiation alone, or a combination of both radiation and BRAF inhibitor. The data showed that combination treatment with radiation and BRAF inhibitor PLX4720 led to a statistically significant survival advantage in these mice, when compared to treatment with vehicle (*p* < 0.0001), PLX4720 (*p* = 0.0098), or radiation (*p* = 0.0059). Combination treatment with PLX4720 and radiation led to a lower tumor proliferation index (Ki67). There was equivalent survival in the combination arms regardless of how radiation was sequenced with the BRAF inhibitor, either concurrently or sequentially (Dasgupta et al., 2012).

These translational studies will inform the next generation of clinical trials of BRAF V600E inhibitors and radiation in patients with BRAF V600E-mutated brain tumors and metastases. At least one of these studies is underway under the umbrella of the Pacific Pediatric Neuro-Oncology Consortium (PNOC, http://www.pnoc.us). This Phase I study will establish the safety and pharmacokinetic characteristics of vemurafenib in children with recurrent or refractory gliomas containing the BRAF V600E mutation. Using the recommended Phase II dose, this trial will proceed to a Phase II study in a pre-surgical cohort of 10 patients requiring debulking surgery at the time of recurrence. These patients will receive neo-adjuvant vemurafenib, allowing measurements of intra-tumoral drug concentrations and target inhibition. An expansion cohort will then be enrolled to estimate efficacy. Children will also be carefully followed for the development of dermal lesions that may be comparable to the keratoacanthomas seen in adults. This trial will start accruing in 2013. If the dose limiting toxicities of vemurafenib in this trial are not prohibitive to its administration in children, then a possible next step will be to provide combination treatment of vemurafenib with concurrent external beam radiation in children with BRAF V600E-mutated high-grade gliomas, or recurrent BRAF V600E-mutated low-grade gliomas.

Resistance to BRAF V600E inhibitors has manifested in the clinic. In BRAF V600E-mutated melanomas, after an initial robust response, there is disease progression within a median of 5–7 months. It is hypothesized that signaling through redundant pathways involving the PI3-kinase (PI3K)/mammalian target of rapamycin (mTOR), VEGF, RAS/RAF/MEK/ERK, and EGFR pathways contributes to such drug resistance (Jang and Atkins, 2013). For example, colon cancers with BRAF V600E mutations do not respond to vemurafenib due to feedback activation of EGFR. Therefore, in pre-clinical studies, vemurafenib is strongly synergistic with EFGR inhibitors like cetuximab for colon cancers expressing BRAF V600E mutations (Prahallad et al., 2012). Whether resistance to BRAF V600E inhibitors also arises in gliomas, where EGFR and PI3K/mTOR pathways are often over-activated, remains to be seen in neuro-oncology patients treated with BRAF V600E inhibitors.

Finally, pediatric oncologists should be cautioned that, with the exception of a single case report (Rush et al., 2013), there is little experience using dabrafenib or vemurafenib in children, and the efficacy of BRAF V600E inhibitors against tumors with the KIAA1549:BRAF molecular fusion is unknown. Additionally, a recent abstract suggests that in glioma cell lines engineered to express the KIAA1549:BRAF fusion, there is resistance to therapy with BRAF V600E inhibitors, and in fact, the paradoxical growth activation (previously only seen in wild-type BRAF cells treated with BRAF V600E inhibitors) is observed (Lang et al., 2012).

## Mammalian Target of Rapamycin

Mammalian target of rapamycin is a serine-threonine kinase in the phosphatidylinositol kinase (PI3K) family that signals downstream of metabolite-sensing pathways, and regulates cellular proliferation, apoptosis, and autophagy (McCubrey et al., 2012). The primary signals activating mTOR are derived from insulin growth factor receptor (IGFR) and the amino acid transporter pathway. Therefore, activation of the mTOR pathway is a reflection of the nutritional status and extracellular stresses in the cellular microenvironment. Many components of the mTORC1 and mTORC2 complexes, in addition to their downstream targets, have been associated with human cancers (Figure 1).

Mammalian target of rapamycin itself forms the catalytic subunit of two protein complexes, mTORC1 and mTORC2. mTORC1 is comprised of several proteins including mTOR, RAPTOR, DEPTOR, PRAS40, and mLST8/GL. This complex is activated by extracellular amino acids that enable mTORC1 to interact with RHEB, the convergence point of amino acid metabolism and insulin signaling. RAPTOR inhibits the kinase activity of mTOR: in high nutrient states, conformational changes occur to expose the mTOR active site to solution. mTOR is then activated as a kinase and can phosphorylate p70-S6 kinase, which then phosphorylates downstream targets like S6 (to form phosphorylated S6, or phospho-S6) and the eukaryotic initiation factor 4E (4EBP1, to form phospho-4EBP1) that increase protein translation and also activate mTOR activity by a positive feedback loop. PRAS40 is a negative regulator of mTORC1, but phosphorylation of PRAS40 by Akt can prevent this inhibition and additionally activate the mTORC1 complex (Manning and Cantley, 2007; Wang et al., 2007).

The mTORC2 complex also responds to nutrient levels and redox states, but with the cellular cytoskeleton as its target. mTOR, RICTOR (rapamycin insensitive companion of mTOR), GβL, and mSIN (mammalian stress-activated protein 1) comprise the mTORC2 complex. A critical function of mTORC2 is activation of Akt (Protein kinase B1) via PDK1 stimulation. Given the role of Akt in diverse cellular processes like glucose metabolism, apoptosis, cell cycle regulation, and transcription, it is not surprising that the mTORC2 complex is an important therapeutic target in cancer therapy (Populo et al., 2012). In an interesting study of regulation of neural stem cell growth Kaul et al. (2012) demonstrate that divergent pathways converge at mTOR to activate growth in gliomas with different driver mutations. In sporadic low-grade gliomas driven by KIAA1549:BRAF and familial low-grade gliomas associated with protein neurofibromin 1 (NF1), mTOR represents a central growth control target. However, the two mechanisms of mTOR activation are distinct in that KIAA1549:BRAF activates mTOR through MEK-dependent tuberin inactivation whereas NF1 loss leads to TORC2-dependent AKT activation. These distinct mechanisms of mTOR activation notwithstanding, mTOR represents a promising therapeutic target in sporadic and NF1 associated low-grade gliomas.

The PI3K/Akt/mTOR axis is also activated in a substantial percentage of pediatric brain tumors. In newly diagnosed pediatric gliomas, 80% of high-grade gliomas and 50% of low-grade gliomas showed enhanced expression of phospho-S6 and phospho-4EBP1, the major downstream targets of mTORC1. Expression of phospho-S6 and/or phospho-4EBP1 has also been associated with a statistically significant decrease in progression-free survival (PFS), regardless of tumor grade (Populo et al., 2012). In low-grade adult brain tumors, activation of the PI3K/mTOR pathway portends shorter overall survival. In a study of 45 newly diagnosed pediatric brain tumors, there was a strong correlation between phospho-S6 expression and decreased survival. There was also a trend toward decreased survival in patients whose tumors expressed phospho-PRAS40. Phospho-S6 expression correlated with PTEN methylation in these tumors as well (McBride et al., 2010). Also, in a molecular analysis study of 92 pilocytic astrocytomas including 43 conventional pilocytic astrocytomas, 25 clinically aggressive/recurrent pilocytic astrocytomas, and 25 anaplastic pilocytic astrocytomas, an increase in cytoplasmic phosphorylated-Akt (phospho-Akt) and phospho-S6 was associated with anaplastic histology (a more aggressive phenotype) and poorer outcomes in pilocytic astrocytomas (Rodriguez et al., 2011). mTOR activation has also been associated with a particular subset of pilocytic astrocytomas called the “low-grade astrocytoma subtype intermediate” (LGSI), found in patients with syndrome neurofibromatosis 1 (NF-1) (Jentoft et al., 2010).

Given the up-regulation of mTOR/PI3K pathway in numerous human cancers, several inhibitors of the mTOR pathway have been developed (Fasolo and Sessa, 2012). The first inhibitor of mTOR was rapamycin or sirolimus, initially developed as an antifungal and then an immunosuppressant for transplant patients. Rapamycin forms a complex with FK506 that in turn interacts with the C-terminus of mTOR and inhibits the mTORC1 complex. Two derivatives of rapamycin, temsirolimus (CCI-779) and everolimus (RAD001, Affinitor), are currently in the cancer clinic. Temsirolimus was FDA-approved to treat mantle cell lymphoma (Samad and Younes, 2010), and everolimus was initially approved to treat renal carcinoma in 2009 (Motzer et al., 2008), as well as subependymal giant cell astrocytomas (SEGAs) in patients with tuberous sclerosis (TSC; see below) (Krueger et al., 2010). Subsequently, everolimus been shown to increase PFS when used in combination with exemestane in patients with advanced breast cancer. Several ATP-competitive mTOR inhibitors are also being tested in the clinic. PP242 is an ATP-competitive inhibitor identified by kinome profiling and has shown activity against both mTORC1 and mTORC2 (Hoang et al., 2012). Other active site inhibitors of mTORC kinase are at different stages of development including Torin-1 and WYE-354 (in pre-clinical studies) and AZD8055, AZD2014, OSI-027, and MLN0128 (in clinical trials) (Laplante and Sabatini, 2012).

Many early mTOR inhibitors were found to have activity against both PI3K and mTOR because of the sequence and structural homology shared by PI3K and mTOR in the ATP-binding cleft. It soon became apparent that such dual inhibitors had “classical hinge kinase binders” as their scaffold, as in XL765 (SAR245409) (Prasad et al., 2011), NVP-BEZ235 (Doghman and Lalli, 2012), GNE-317 (Salphati et al., 2012), OSI-027 (Bhagwat et al., 2011), and SF1126 (Mahadevan et al., 2012). These dual inhibitors can potently inhibit multiple downstream pathways and induce apoptosis in a number of cancers from different tumor tissues. The majority of these dual inhibitors are lipophilic, necessitating the development of a second generation of these compounds to overcome solubility issues by linking rapamycin to a PI3K inhibitor with a soluble linker. Many of these compounds are currently in clinical trials for various solid tumors (Molckovsky and Siu, 2008).

Can inhibitors of the mTOR/PI3K axis be used to treat gliomas? As outlined above, the PI3K/mTOR pathway is activated in over half of low-grade gliomas. Pre-clinical data from our laboratory strongly support a role for PI3K/mTOR inhibitors in adult and pediatric low-grade gliomas. Recently published studies show that XL765, a dual PI3K/mTOR inhibitor, has potent activity in an intracranial xenograft mouse model of high-grade glioma. Furthermore, XL765 also shows synergistic activity with temozolomide in high-grade glioma xenografts (Prasad et al., 2011) and is currently under evaluation in Phase I clinical trials for adult brain tumors, in combination with temozolomide (Nghiemphu et al., 2010).

Additionally, pre-clinical data from our laboratory were one of the first to demonstrate a correlation between PTEN expression, Akt activity, and glioma histology in adults. More aggressive gliomas like glioblastomas had decreased levels of PTEN, which correlated with higher levels of Akt activity. In adult low-grade gliomas, there was also decreased PTEN expression, without discernible PTEN losses or mutation, and this decreased PTEN expression was associated with increased activity of the Akt pathway (Ermoian et al., 2002). Interestingly, another brain tumor that also demonstrates this molecular signature of mTOR activation is TSC-associated SEGA. In a landmark series of 28 patients aged 3 years or greater with SEGAs, tumors showed an unprecedented response in 75% of patients treated with everolimus. The duration of response was over 3 months, no other therapy was required, and patients experienced symptomatic relief of their seizure frequency. The most common adverse effects were stomatitis, upper respiratory tract infections, sinusitis, otitis media, and pyrexia. Grade 3 adverse events were noted in 36% of patients and included infections, stomatitis, and convulsions (Krueger et al., 2010).

These data from TSC suggest that low-grade gliomas with mTOR activation may also benefit from treatment with everolimus. Therefore, researchers at University of California San Francisco have started a Phase II trial of everolimus for the treatment of adults with recurrent low-grade gliomas (Haas-Kogan et al., 2013). Between 2009 and 2012, 36 patients with low-grade gliomas have been enrolled. Histologies at original diagnosis were oligodendroglioma (*n* = 14), astrocytoma (*n* = 9), and oligoastrocytoma (*n* = 13). However, at time of recurrence (prior to study enrollment), 9 of the 36 patients had transformed to higher-grade disease. Prior to treatment with everolimus, 11 patients had received radiation and 23 had received temozolomide. Currently, 11 patients continue on active therapy with everolimus, 15 have progressive disease, and the remaining discontinued drug due to toxicity (*n* = 2), poor follow-up (*n* = 1), refusal of further therapy (*n* = 4), or unrelated inter-current disease (*n* = 2). Encouragingly, 15 patients had stable disease for over a year, and four had stable disease for over 2 years while on treatment, all despite multiple prior recurrences (Haas-Kogan et al., 2013).

Additionally, our laboratory is currently seeking to exploit recent findings that suggest crosstalk between the BRAF and PI3K/mTOR signaling pathways as driving mutations in pediatric gliomas. In a recent study performed on neural stem cells, cross talk between the KIAA1549:BRAF fusion protein and the mTOR pathway has been demonstrated. The neural stem cell proliferation secondary to the BRAF fusion protein is actually mediated by hyper-activation of the mTOR pathway, suggesting crosstalk between these two critical pathways in pediatric gliomagenesis (Kaul et al., 2012). Given the prevalence of activation of the PI3K/mTOR axis and BRAF V600E mutations in pediatric tumors, we designed a pre-clinical study to determine whether inhibitors of mTOR and of BRAF V600E cooperate to enhance cytotoxicity specifically in pediatric low-grade gliomas. In an *in vivo* flank model of a pediatric pilocytic astrocytoma, subcutaneous xenografts of BT40 (which carries a BRAF V600E mutation) were treated with vehicle, PLX4720, everolimus, or combination of PLX4720 + everolimus, and the outcome measures of tumor size and animal survival were followed. In murine xenografts of a pilocytic astrocytoma with mutated BRAF V600E, treatment with PLX4720 + everolimus led to a statistically significant survival advantage, when compared to treatment with vehicle alone (*p* = 0.0002), PLX4720 alone (*p* = 0.0126), or everolimus alone (*p* = 0.0031). Treatment with PLX4720 + everolimus also led to significantly smaller tumors when compared to treatment with vehicle alone (*p* < 0.0001), PLX4720 alone (*p* < 0.0001), or everolimus alone (*p* = 0.0082). This is the first *in vivo* demonstration of combinatorial activity of an mTOR inhibitor with a BRAF inhibitor in gliomas, and will inform future clinical trials in pediatric brain tumor patients (Dasgupta et al., 2013).

## Angiogenesis

Angiogenesis is a tightly regulated process by which novel vasculature forms in the brain. In brain tumors, this process enables transformation to a more vascular or angiogenic phenotype. Gliomas are hyper-vascular tumors, and routinely overexpress pro-angiogenic factors like VEGF, EGF, PDGF, FGF, SDF-1, Tie2, and TGF-beta. In fact, the PI3K/Akt pathway, described above, also increases angiogenesis by up-regulation of VEGF and HIF1α (Jensen, 1998). In gliomas, there is evidence that p53 mutations significantly increase the mean vessel density of pilocytic astrocytomas, and affect important regulators of angiogenesis including thrombospondin-1, serpin E1, and MMP-9 (Gaiser et al., 2009). Given the utility of bevacizumab (Avastin, an inhibitor of VEGF-A) in the clinic to treat recurrent gliomas (Sweet et al., 2012), inhibitors of angiogenesis have become an attractive target in the neuro-oncology clinic (Butowski, 2011).

Angiogenesis may also correlate with brain tumor prognosis. Nearly two decades ago, contrast enhancement in low-grade gliomas was recognized to portend poor outcomes in patients (Piepmeier, 1987). The density of blood vessels is a prognostic indicator for patients with gliomas: higher blood vessel density correlates with poorer survival (Leon et al., 1996). Some studies have suggested that low-grade gliomas integrate existing vasculature in the brain, while glioblastomas become hyper-vascular by angiogenesis (Folkerth, 2000). More recently, an interesting MRI study performed in 46 adults with low-grade gliomas showed that both microvascular leakage and contrast enhancement in low-grade gliomas were associated with the lowest PFS (Piepmeier, 1987; Dhermain et al., 2010).

Pilocytic astrocytomas are particularly vascular tumors, and therefore there is great interest in targeting angiogenesis in these low-grade gliomas. Researchers have also examined KIT expression in 35 pilocytic astrocytomas and 45 other pediatric brain tumors using immunohistochemistry. They found that 35% of pilocytic astrocytomas express KIT, as do some of the normal brain specimens. KIT was also found in the endothelial cells of pediatric brain tumors, and was more common in pilocytic astrocytomas diagnosed at a young age (Puputti et al., 2010). In a study comparing the vasculature of 59 pilocytic astrocytomas to that of 62 adult glioblastomas, researchers examined the vessel maturity, vessel turnover, and VEGF expression between the pilocytic astrocytomas and glioblastomas. They found that pilocytic astrocytomas had less turnover of blood vessels, and had larger but fewer blood vessels than glioblastomas. There was a correlation between the decreased number of vessels and higher expression of VEGF-A, suggesting that pilocytic astrocytomas may overexpress this protein. The authors suggested that VEGF-A might serve as a druggable target in pilocytic astrocytomas (Sie et al., 2010).

Based on these studies of angiogenesis in pediatric tumors, a Phase I dose escalation study of sunitinib, a VEGF inhibitor, was conducted in 23 children aged 2–21 with various refractory solid tumors. Sunitinib was administered in daily 28-day cycles, with a 14-day break between cycles. The major toxicities of sunitinib in children were neutropenia, thrombocytopenia, transaminopathy, and fatigue. Two of 23 patients developed decreased cardiac ejection fraction. However, encouragingly, one patient with diffuse intrinsic pontine glioma (DIPG) and one patient with a ganglioglioma had stable disease for 2–9 cycles. They found that a significant correlation between treatment with sunitinib and decreased plasma levels of endoglin, a marker of tumor-associated endothelial cells. This result suggests that following endoglin levels might be used in pharmacodynamic monitoring of sunitinib treatment in children (Dubois et al., 2011). There has also been a study in children with recurrent high-grade gliomas in which patients received a combination of cytotoxic chemotherapy (mostly with irinotecan) and bevacizumab. The combination therapy was very well tolerated, and the greatest benefit observed in tumors with the highest contrast enhancement (Parekh et al., 2011). A Phase II study of sorafenib was opened for pediatric patients with low-grade astrocytomas. This study has temporarily been suspended, reportedly due to excess progressive disease (Clinical Trials NCT01338857), but we currently await the publication of the interim results of this trial (Karajannis, 2011).

Progression of gliomas after treatment with bevacizumab has been reported. In fact, certain reports suggest that treatment of high-grade gliomas with bevacizumab has precipitated a more invasive and diffuse phenotype of gliomatosis cerebri. Treatment with bevacizumab may also increase the rate of gliomatosis cerebri, when compared to treatment of high-grade gliomas with conventional therapies alone. However, it remains to be seen if these trends are borne out in the data from large randomized clinical trials and meta-analyses (Wick et al., 2011). Another retrospective study of 110 patients with high-grade gliomas treated with bevacizumab suggests that treatment with low-dose bevacizumab is associated with a favorable prognosis (Lorgis et al., 2012). In children with low-grade gliomas treated with bevacizumab, diffuse recurrence patterns were not seen. Patterns of progression must be closely followed in future trials, and molecular mechanisms of recurrence may further influence combination therapies of angiogenesis inhibitors with other targeted inhibitors (Couec et al., 2012).

In addition to bevacizumab and other targeted VEGF inhibitors (e.g., sorafenib, sunitinib), new anti-angiogenics are also being developed (Arbab, 2012). Thalidomide, a derivative of glutamic acid, was initially developed as a sedative, and later was discovered to have an immuno-modulatory role as well as anti-angiogenic properties (Kumar and Chhibber, 2011). Thalidomide was mostly withdrawn from clinical use secondary to its well-characterized teratogenic effects. Later it became a mainstay of treatment in multiple myeloma, where it has shown response in more than 30% of patients with refractory disease (Moehler, 2012). The major side effects of thalidomide are hematologic. It causes venous thromboembolism and bone marrow suppression, as well as peripheral neuropathy and secondary malignancies. The immunomodulator functions of thalidomide and its analogs are mediated by inhibiting TNF-αs. The anti-angiogenic properties of thalidomide are mediated by down-regulating signaling from the VEGF receptor and the FGF receptor (Shortt et al., 2013).

Early experiences with combining radiation with thalidomide in both adult and pediatric gliomas have been discouraging. In Radiation Therapy Oncology Group (RTOG) 9806, a single arm Phase II study instituted by the RTOG, patients with high-grade gliomas were treated with concurrent radiation therapy and thalidomide (Alexander et al., 2013). There was no overall survival benefit to this combination treatment, with a median survival of 10 months, when compared to historical controls. When these results are compared to the long-term results of the randomized trial of radiotherapy versus radiotherapy and temozolomide as published by Stupp et al. (2005) the median survival is lower than that of the patients treated with radiotherapy alone (12 months) and with concurrent temozolomide and radiotherapy (14.6 months). This discrepancy in results may be associated with the age of patients permitted to enroll (as the Stupp study did not permit patients over 70 years of age) or increased toxicity associated thalidomide, especially venous thrombosis (Warren et al., 2011). Similarly, in a Phase II study combining thalidomide and radiation therapy for pediatric brain stem gliomas and glioblastomas, survival was not improved by the addition of thalidomide (Turner et al., 2007). Similarly, no survival advantage has been seen in glioblastomas patients treated adjuvantly with combinations of thalidomide with other agents (after surgery, radiation, and temozolomide) including thalidomide with temozolomide; thalidomide, and temozolomide with celecoxib (Riva et al., 2007; Kesari et al., 2008); thalidomide with procarbazine (Ruiz et al., 2012); and thalidomide with irinotecan (Giglio et al., 2012). However, promising early results have been recently been reported in adult glioblastoma patients treated with adjuvant temozolomide, thalidomide, isotretinoin with/without celecoxib (Gilbert et al., 2010). Longer follow-up is necessary.

Children with brain tumors treated with thalidomide may fare better. In a study by the Korean Society for Pediatric Oncology in 12 children with DIPGs treated with radiation, followed by temozolomide and thalidomide, the response rate was over 80%, the 6 month progression-free survival was 58.3% and the overall survival was 12.7 months. The regimen was well tolerated by children with hematological toxicity being the major adverse outcome. These results must be replicated in larger randomized studies (Kim et al., 2010).

An analog of thalidomide, lenalidomide (CC-5013) is thought to be more potent in its anti-inflammatory properties than thalidomide, with a more favorable side effect profile (Shortt et al., 2013). Its higher potency has led to several trials in which lenalidomide is currently under investigation as an anti-cancer agent, including multiple myeloma (Moehler, 2012), Crohn’s disease (Mansfield et al., 2007), and other solid tumors (Segler and Tsimberidou, 2012). When lenalidomide was evaluated in adults with recurrent primary CNS tumors in adults, as with thalidomide, these tumors showed no objective response to treatment with lenalidomide but instead increased hematological toxicity (Fine et al., 2007). However, in PBTC-018, a phase I trial of 51 pediatric patients with recurrent, refractory, or progressive CNS tumors, objective responses were seen primarily in children with low-grade gliomas. Lenalidomide was found to be tolerable in children with brain tumors at doses of 116 mg/m^2^/day, with the most commonly observed toxicity being myelosuppression (Warren et al., 2011). Lenalidomide is also being tested in combination with radiation for pediatric DIPGs in an open National Cancer Institute-sponsored clinical trial (NCI 10-C-0219).

## Summary

Multiple diverse pathways drive gliomagenesis, and their convergence causes low-grade gliomas to develop, persist, and recur after treatment. With increasing understanding of the molecular underpinnings that drive brain tumor formation, novel targeted strategies are being developed to combat this disease. In the treatment of low-grade gliomas, which have a long natural history, resistance to therapies is common. This review summarizes three pathways that are the subject of current pre-clinical and clinical studies. With the explosion of new targeted inhibitors in the cancer clinic, each one of these pathways can be exploited individually or in combination with other inhibitors or radiation to develop rational combination treatments for gliomas.

## Conflict of Interest Statement

The authors declare that the research was conducted in the absence of any commercial or financial relationships that could be construed as a potential conflict of interest.
